# Mid-infrared III–V semiconductor lasers epitaxially grown on Si substrates

**DOI:** 10.1038/s41377-022-00850-4

**Published:** 2022-06-01

**Authors:** Eric Tournié, Laura Monge Bartolome, Marta Rio Calvo, Zeineb Loghmari, Daniel A. Díaz-Thomas, Roland Teissier, Alexei N. Baranov, Laurent Cerutti, Jean-Baptiste Rodriguez

**Affiliations:** grid.461998.b 0000 0004 0390 3782 IES, University of Montpellier, CNRS, 34000 Montpellier, France

**Keywords:** Semiconductor lasers, Silicon photonics

## Abstract

There is currently much activity toward the integration of mid-infrared semiconductor lasers on Si substrates for developing a variety of smart, compact, sensors based on Si-photonics integrated circuits. We review this rapidly-evolving research field, focusing on the epitaxial integration of antimonide lasers, the only technology covering the whole mid-to-far-infrared spectral range. We explain how a dedicated molecular-beam epitaxy strategy allows for achieving high-performance GaSb-based diode lasers, InAs/AlSb quantum cascade lasers, and InAs/GaInSb interband cascade lasers by direct growth on on-axis (001)Si substrates, whereas GaAs-on-Si or GaSb-on-Si layers grown by metal-organic vapor phase epitaxy in large capability epitaxy tools are suitable templates for antimonide laser overgrowth. We also show that etching the facets of antimonide lasers grown on Si is a viable approach in view of photonic integrated circuits. Remarkably, this review shows that while diode lasers are sensitive to residual crystal defects, the quantum cascade and interband cascade lasers grown on Si exhibit performances comparable to those of similar devices grown on their native substrates, due to their particular band structures and radiative recombination channels. Long device lifetimes have been extrapolated for interband cascade lasers. Finally, routes to be further explored are also presented.

## Introduction

The mid-infrared (MIR) wavelength range is often defined as the 2–12 µm range, i.e., photon energies between 0.62 and 0.1 eV. It includes several transparency windows of the atmosphere and the fingerprint absorption lines of many molecules^1^. It is thus well suited for a variety of high societal impact photonic sensors or devices, and MIR optoelectronics and sensors have attracted much research efforts in the past decade^2^. However, these sensors are still ensembles of discrete components, which makes them bulky and expensive and limits their widespread use, in spite of ever-increasing societal demand.

Silicon photonics has recently emerged in the data/telecom field as the key enabling technology to fabricate—through various approaches—low-cost, chipscale devices thanks to mature CMOS processes and large silicon wafer size^3–6^. This technology can straightforwardly be adapted to the short-wave MIR (*λ* < 3 µm) where silicon, Si_3_N_4_, and SiO_2_ are transparent^7^, whereas extension to the whole MIR is possible through the integration of other designs or materials^8–11^. MIR photonic integrated circuits thus appear as a promising approach to develop low-cost, compact sensors, provided efficient laser sources can be integrated with the circuit. Still, the development of such systems requires the use of on-axis (001) silicon substrates—i.e., exhibiting a residual miscut angle lower than ∼0.5°—to benefit from the large-scale, low-cost industrial Si technology.

Heterogeneous integration is the most advanced strategy nowadays and bonded MIR diode^12–15^, interband cascade^16^, and quantum cascade^17–19^ lasers have been demonstrated. Whatever the application, however, there is evidence that the direct epitaxy of the III–V semiconductor laser heterostructures on Si could surpass the heterogeneous strategy on a mid- to long-term basis^20^. In addition, it uses and wastes much less III–V materials, which makes it more environmental friendly than the heterogeneous approach. Much work has thus been done recently on the epitaxial integration of InAs/GaAs or InAs/InP quantum dot lasers (QDLs) on Si substrates for data/telecom applications in the near-infrared^21–25^. In the MIR spectral range, the most efficient semiconductor lasers are InP-based quantum-cascade lasers (QCLs)^26^, InAs-based QCLs^27^, GaSb-based interband diode lasers (DLs)^28^, and InAs/GaInSb interband cascade lasers (ICLs)^29,30^. In spite of excellent performances when grown on their native InP substrate, InP-based QCLs grown on Si substrates exhibit until now only poor performances^31,32^, probably due to a too-low overall heteroepitaxial-material quality at this stage. In contrast, InAs-based QCLs^33,34^, GaSb-based DLs^35–37^, and InAs/GaInSb ICLs^38^ grown on Si showed promising results. These devices belong to the so-called “antimonide” technology that we review now.

## The antimonide technology

**Fig. 1:**
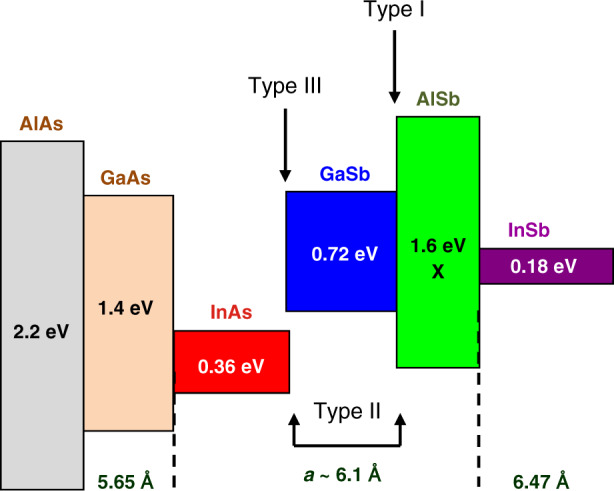
Band alignment at III-As and III-Sb interfaces. The boxes represent, to scale, the bandgap of the compounds, whereas the top and bottom lines indicate the position of the conduction- and valence-band, respectively. The lattice parameters of GaAs, GaSb and InSb are indicated on the horizontal axis. The arrows show examples of band alignments

The antimonide semiconductors refer to the Sb-rich III–V compounds that are usually grown on GaSb or InAs substrates with a lattice constant close to 0.61 nm. This includes GaSb, InAs, and AlSb compounds and their ternary, quaternary, or even pentanary alloys. They span a large bandgap range—from ∼0.1 to ∼1.8 eV—while being closely lattice-matched to InAs or GaSb. A very unique feature of this technology among III–V semiconductors^39^ is the variety of accessible band alignments (Fig. 1). The type-I alignment with electrons and holes confined in the same material, typical for arsenides heterostructures, exists with antimonides (e.g., AlGaSb/GaSb, GaSb/GaInSb). However, type-II and type-III alignments are also possible. Indeed, with the valence-band maximum of AlSb being lower than the conduction-band minimum of InAs, the AlSb/InAs system is type-II, whereas the conduction-band minimum of InAs being lower than the valence-band maximum of GaSb, the InAs/GaSb system is type-III, also known as staggered type-II (Fig. 1). On one hand, this results in giant conduction-band offsets at the InAs/AlSb interface and on the other hand this allows adjusting the effective bandgap of InAs/GaSb superlattices simply by changing the layer thicknesses.

In addition, III-Sbs exhibit high carrier mobilities. InSb and GaSb have, respectively, the highest electron and hole mobilities among all compound semiconductors. These properties make them particularly attractive for low-energy, high-speed transistors^40^. InAs also has the second-smallest electron effective mass, an important asset since the gain in QCLs is inversely proportional to the 3/2-power of this parameter^27^.

III-Sb compounds are thus unique among III–V semiconductors, and they allow the designing of man-made artificial materials with effective bandgaps spanning a huge wavelength range, from the near-infrared (IR) to the far-IR and THz^41^.

These impressive properties however do not come for free. At wavelengths shorter than 3 µm, MIR lasers rely on complex alloys such as GaInAsSb QWs and AlGa(In)AsSb barrier layers. Their composition, and in particular the As/Sb ratio, should be tightly controlled to avoid relaxation within the laser structure itself. This is challenging since the As and Sb group-V species compete to incorporate into the alloys, and this competition is sensitive to a number of parameters such as the absolute value of each group-V flux, the As/Sb flux ratio, the substrate temperature, and the alloy growth rate^42^. Detailed calibrations should thus be performed for each set of growth conditions. On another ground, the InAs/AlSb type-II and InAs/GaSb type-III heterostructures exhibit no-common atom interfaces. A variety of configurations can thus result from the growth sequence. The bonds at the interface between the two base layers can be of (Al,Ga)–As type, In–Sb type, or any combination of both types, leading to localized, from high-tensile to high-compressive, strain. For devices based on short-period superlattices such as QCLs or MIR photodetectors, the interfaces affect not only their structural quality but also their electronics and optical properties^43^. Interfaces have thus to be taken into account in the device design. In turn, they can be considered and used as additional tools to fine-tune the band structure.

Finally, it is worth mentioning that until now molecular-beam epitaxy (MBE) is the only technique allowing the growth of laser-quality III-Sbs heterostructures^44^, again in marked contrast to most other III–V technologies that heavily rely on metal-organic vapor-phase epitaxy (MOVPE). This comes from high Al-contents in the layers, complex alloys, and typical low growth temperatures of these compounds. Although high-performance far-IR InAs/GaSb photodetectors have recently been grown by MOVPE^45^, efficient antimonide lasers grown by MOVPE remain to be demonstrated.

We focus the following of this review article on the epitaxial integration of MIR antimonide lasers on Si substrates.

## Epitaxial growth of antimonide heterostructures on (001)Si substrates

All large scale Si foundries working with on-axis (001)Si substrates, much work has been devoted in the past decades toward growing high quality III–V optoelectronic devices on such substrates. However, the III–V-on-Si epitaxial layers are generally plagued by high densities of micro-cracks, dislocations, or antiphase boundaries^24,32,46–50^, all detrimental defects that should be mitigated or avoided whenever possible.

Microcracks may appear because of the different thermal expansion coefficients of III–Vs and Si materials. This can be avoided by adapting the heating/cooling rates during growth, and by keeping the whole heterostructure thickness below a critical value, estimated to be around 10 µm for GaSb-based DLs (see section “MBE-grown GaSb laser diodes on MOVPE-on-Si templates”).

Dislocations arise from strain relaxation when growing a layer with a lattice constant different than that of the substrate, and are thus unavoidable. Since they act as non-radiative recombination centers, the density of threading dislocations should in principle be reduced to limit device degradation^51,52^. When GaSb is grown on substrates such as GaAs or Si, the large lattice-mismatch (8 or 12%, respectively) induces the formation of an interfacial network of edge-type misfit dislocations^53–57^ that provides the most efficient strain-relaxation pathway^58^. However, dislocation arms still thread through the whole heterostructure. Various strategies have proved efficient to reduce threading dislocation densities (TDDs) down to the 10^6^ cm^−2^ range for InAs/GaAs QDLs grown on Si^59–63^. However, they have not been implemented yet in the growth of III-Sbs on Si and TDDs are typically in the 10^7^–10^8^ cm^−2^ range after a couple of micrometers of growth.

**Fig. 2:**
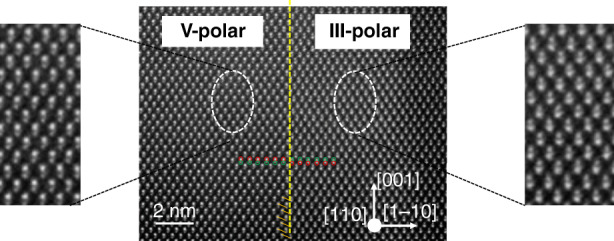
Scanning transmission electron microscopy high-angle annular dark-field image of a GaSb layer grown on an on-axis (001) Si substrate. The dashed yellow line indicates an APB separating two polar domains. The insets show the Ga-Sb dumbbells in each domain, where the brighter atom is Sb due to its higher atomic number. Adapted with permission from ref. ^93^. © John Wiley & Sons

Antiphase domains (APDs) form when growing III–V materials on on-axis (001)Si due to the fact that Si has a non-polar diamond crystal structure whereas zinc blende III–V compounds have a polar crystal structure. The growth of a zinc blende material on a diamond material allows the simultaneous formation of two different crystal phases^64,65^. These domains are separated by antiphase boundaries (APBs) that are two-dimensional defects made of III–III and V–V bonds (Fig. 2), generating a local excess or lack of electrical charges.

Consequently, APBs create conduction paths^66^, and they should not cross the epitaxial stack. Various strategies have been implemented to avoid the formation of polar domains or to avoid threading APBs. The most straightforward one is to use (001)Si substrates intentionally miscut toward the [110] direction with an angle >4°^64,67–72^. Combined with high-temperature annealing, the stable Si surface is then populated with a majority of double steps that avoid APD formation^64^. This large miscut angle however is not compatible with industrial standards. Various on-axis (001)Si substrates preparations and III–V nucleation stages^73–83^, or the use of patterned Si substrates^84–90^, have thus been specifically developed to avoid threading APBs for GaAs or GaP epitaxy on industry-compatible substrates. Details on these procedures are beyond the scope of this article but can be found in the corresponding references.

**Fig. 3:**
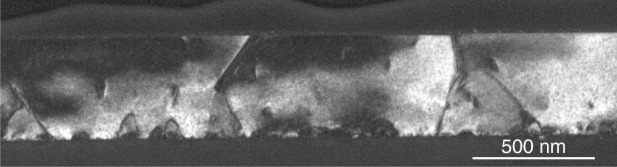
Dark-field cross-section TEM image of 500-nm-thick GaSb layer on 0.5-degree miscut Si (001) showing the burying of the APDs. Other defects such as dislocations are also visible. Reprinted with permission from ref. ^93^. © John Wiley & Sons

Regarding the epitaxial integration of antimonide lasers on Si substrates, until 2020 all devices were grown on (001)Si substrates exhibiting a 4-to-7°-miscut angle with respect to the nominal (001) orientation^91,92^, not compatible with Si-photonics platforms. We have recently investigated in detail the MBE growth of GaSb layers on on-axis (001)Si substrates, and we have demonstrated that careful optimization of both the Si surface preparation and the GaSb nucleation stage results in one polarity domain, the APD, being overgrown by the other domain, so-called the main polarity domain (Fig. 3)^93^. The [110] direction of the residual miscut and high-temperature annealing of the Si substrate prior to epitaxy are key points that promote a well-ordered organization of the steps at the Si surface^93^. In turn, the different incorporation rates of group-III elements at step edges result in the main polar domains growing faster than the APDs, and thus in the burying of the latter^94^. The GaSb thickness needed to bury the APDs varies with the residual miscut angle. It is typically around 250, 500, and 1000 nm for residual miscuts of 1, 0.5, and 0.2, respectively^93^. Such GaSb-on-Si layers can then be used as templates for further growth^95^.

Another interesting option in view of future large-scale production is to decouple APD management and device growth. In particular, MOVPE recently proved efficient to prepare large-size on-axis Si substrates with the step organization needed to avoid—or bury—APDs with different III–V materials systems^49,75–77,96^. Demonstrations were even reported for Si substrates up to 300-mm diameter^76,77,96^. InAs/GaAs QDLs grown by MBE are now routinely developed on MOVPE GaP-on-Si templates^60^. In that vein, it has been recently demonstrated that a dedicated Si-surface preparation coupled to III–V MOVPE growth in a cluster tool adapted from the microelectronics industry allows APDs burying after a few-hundreds-nm GaSb on 300-mm on-axis (001)Si wafers^96^. Yet another possibility is the growth of antimonide devices on threading-APB-free GaAs-on-Si templates^78^.

In the following, we review the literature, and in particular our effort on integrating antimonide lasers on various III–V-on-Si templates.

## GaSb-based laser diodes grown on Si

### Laser diode heterostructure

**Fig. 4:**
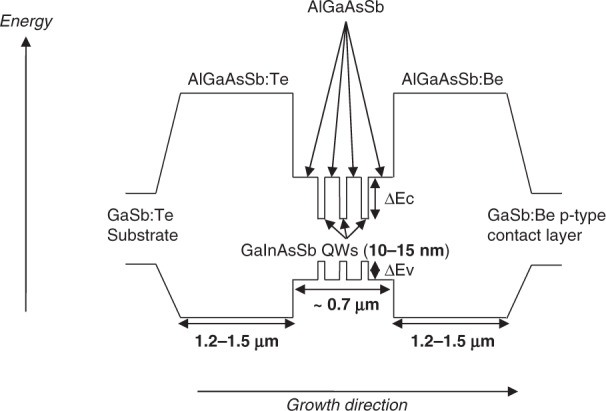
Typical band structure of a type-I QW GaInAsSb/AlGaAsSb diode laser emitting in the 2–3 μm wavelength range. The materials used in the different layers are indicated together with their typical thickness

GaSb-based MIR DLs are based on the GaInAsSb/AlGaAsSb quantum-well (QW) material system. The cladding and barrier layers are made of Al-rich, wide band-gap and low refractive index, AlGaAsSb compounds whereas the emitting QWs are based on the GaInAsSb narrow-gap alloy^97^. These DLs proved efficient in the whole 1.5–3.3 µm wavelength range. A typical band profile for such a laser heterostructure is shown in Fig. 4 together with the layer thicknesses. The active zone is made of strained GaInAsSb QWs (typically 2–3 QWs) confined by lattice-matched AlGaAsSb barrier layers. The waveguide and barrier layers are made of AlGaAsSb alloys with Al and As contents around 25–35% and 3–4%, respectively, whereas the Al and As contents are increased to 70–90 and 6–8%, respectively, in the cladding layers. Graded-AlGaAsSb layers are inserted below the bottom cladding layer and above the top cladding layer to gradually evolve from/toward GaSb. All AlGaAsSb layers are lattice-matched to GaSb, whereas the GaInAsSb QWs exhibit a ∼1.5% lattice mismatch with respect to GaSb. Typical In and As contents are in the 30–40 and 6–9% ranges, respectively. Obviously, the alloy compositions and QW thicknesses are chosen according to the target emission properties. The cladding layers are doped in the 10^18^ cm^−3^ range, whereas the waveguide and the active zone are not intentionally doped, which results in residual *p*-type doping in the 10^16^ cm^−3^ range^44^. The *p*-type doping level is progressively increased up to the 10^19^ cm^−3^ range when growing the GaSb:Be contact layer. More information on the properties of such laser diodes grown on GaSb substrates can be found in refs. ^28,98,99^.

### All-MBE grown GaSb-on-Si laser diodes

Optically pumped lasers have been demonstrated with AlGaSb/GaSb/AlGaSb double heterostructures grown by MBE on Si as early as 1986^100^, but twenty more years were needed before the demonstration of the first electrically pumped DLs^91^. (001) Si substrates with a 5° miscut-angle toward the [110] direction were used to reduce the formation of APDs. The structure, designed for an emission near 1.5 µm, was based on 10-nm wide GaSb QWs confined by Al_0.3_Ga_0.7_Sb barrier layers^91^. Pulsed-operation lasing was achieved at 77 K only. Continuous wave (CW) operation at or above room temperature (RT) remained challenging for a few years. We first demonstrated pulsed RT operation with a laser structure similar to that usually grown on GaSb substrates (cf. Fig. 4), but grown on an *n*-type Si substrate with a 4° miscut-angle toward the [110] direction^101^. The active region composed of two 11-nm-wide Ga_0.65_In_0.35_As_0.06_Sb_0.94_ QWs was confined by Al_0.35_Ga_0.65_As_0.03_Sb_0.97_ barrier layers and embedded in between 1.5 µm-thick Al_0.9_Ga_0.1_As_0.0.7_Sb_0.93_ *n*- and *p*-type cladding layers. The threshold current density was around 1.5 kA cm^−2^ and the laser peak emission at 2.33 µm. This is ∼15 times higher than for similar lasers grown on GaSb substrates^102^ and is most probably due to carrier and/or optical losses related to crystal defects.

A first breakthrough—still on high-angle (7°) miscut Si substrates—occurred thanks to a specially developed ex situ Si substrate preparation^103^ and to a new process where both contacts were taken on the epitaxial side to avoid that the current flows across the III-Sb/Si interface^35^. An InAs_0.92_Sb_0.08_ layer lattice-matched to GaSb was inserted within the buffer layer. It exhibits a very low contact resistance and a high electrical conductivity as compared to GaSb and can be used as an etch-stop layer during device processing, as well as the bottom-contact layer^104^. The laser structure was designed to emit at 2 µm with two 9-nm-wide Ga_0.65_In_0.35_As_0.05_Sb_0.95_ QWs confined by 30 nm Al_0.25_Ga_0.75_As_0.02_Sb_0.98_ layers. Threshold current densities around 900 A cm^−2^ were measured in pulsed mode at RT with 100 µm × 1.4 mm Fabry–Perot cavities. CW operation was demonstrated with 8 µm × 2 mm cavities, which was not possible when the current crossed the III-Sb/Si interface. Several mW/facet output power was obtained at temperatures up to 40 °C. The so-called characteristic temperature *T*_0_ that characterizes the evolution of the threshold current with the temperature, varied from 80 K below 20 °C to 40 K at higher temperatures.

**Fig. 5:**
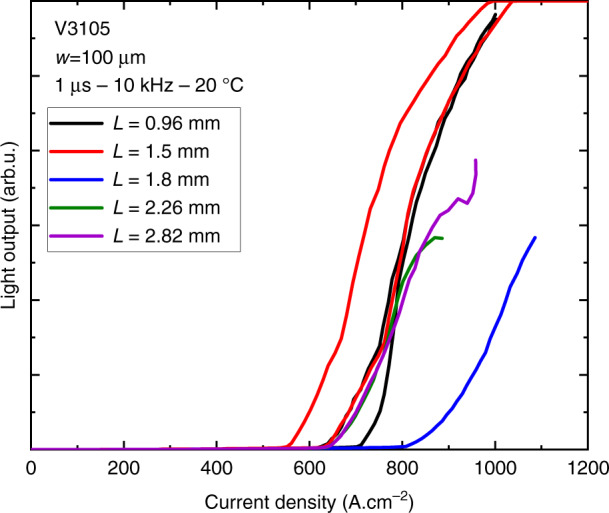
*L*–*I* curves in pulsed mode from various broad are GaSb DLs grown on a Si substrate with a 0.18° residual miscut angle. DLs with 100-µm wide ridges and various cavity lengths L were tested under pulsed conditions at 20 °C

In spite of indisputable progress, these DLs were still grown on large-miscut substrates, incompatible with PICs development. The second breakthrough came when we demonstrated the ability to grow GaSb layers on on-axis Si substrates thanks to APD burying within a buffer layer^93^. More details are given in the section “Epitaxial growth of antimonide heterostructures on (001)Si substrates” above and in ref. ^93^. Detailed results for DLs grown on substrates with a 0.5° residual-miscut angle were reported in ref. ^37^. Threshold current densities in the pulsed regime were in the 400–500 A cm^−2^ range for broad area DLs, whereas narrow-ridge devices operated in CW up to 80 °C (set-up limited) with 4–10 mW/facet output power in this temperature range, and a *T*_0_ characteristic temperature around 120 K. Another series of DLs grown on a substrate with a residual-miscut angle as low as 0.18° toward the [110] direction (measured by X-ray diffraction) exhibited a threshold current density in the 550–800 A cm^−2^ range (Fig. 5). The difference in the thresholds of DLs grown on 0.5° (ref. ^37^) and 0.18° (Fig. 5) residual-miscut (001)Si substrates can be ascribed to different residual threading dislocation densities in these laser structures (cf. “Summary”).

**Fig. 6:**
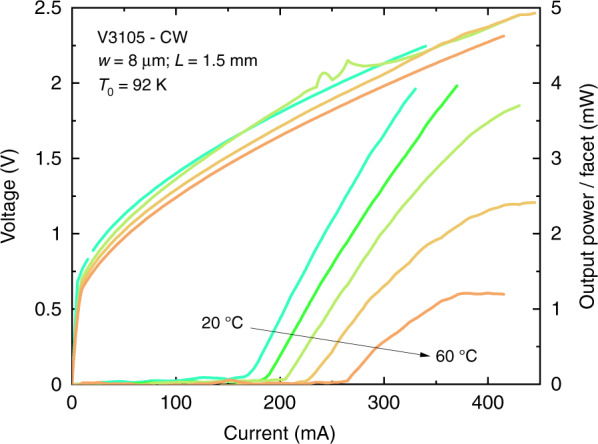
*L*–*I*–*V* curves in CW at various temperatures for a narrow-ridge GaSb DL grown on a Si substrate with a 0.18° residual miscut angle. The DL was tested in CW from 20 °C to 60 °C, with 10 °C steps

**Fig. 7:**
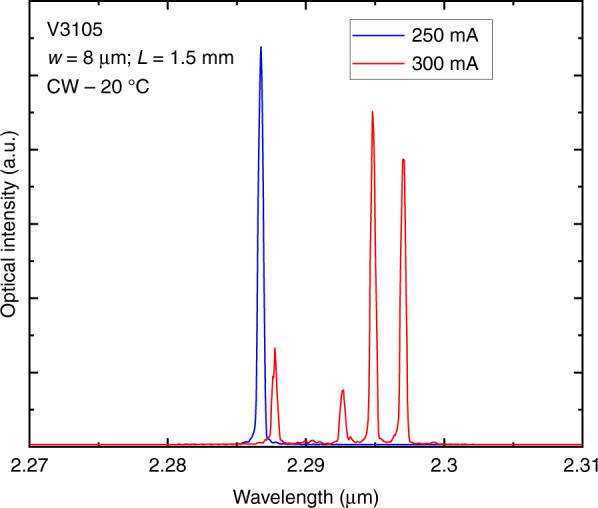
Emission spectra taken in CW at room temperature from a 8 µm × 1.5 mm GaSb DLs grown on a Si substrate with a 0.18° residual miscut angle. The wavelength shift is due to the different drive currents (250 and 300 mA) that induce different internal temperature of the DL

We show in Fig. 6 the temperature-dependent data for a 8 × 1500 µm^2^ DL grown on 0.18° residual-miscut (001)Si substrates. CW operation was readily achieved up to 60 °C with 1–5 mW/facet output-power and *T*_0_ ∼90 K. Typical emission spectra taken at room temperature from a DL driven at 1.25 × *I*_th_ (250 mA) and 1.5 × *I*_th_ (300 mA) are displayed on Fig. 7. The emission was near 2.3 µm, as expected from the structure design. It shifted toward a longer wavelength at a rate of ∼0.16 nm mA^−1^, a useful property in view of sensing applications.

### MBE-grown GaSb laser diodes on MOVPE-on-Si templates

**Fig. 8:**
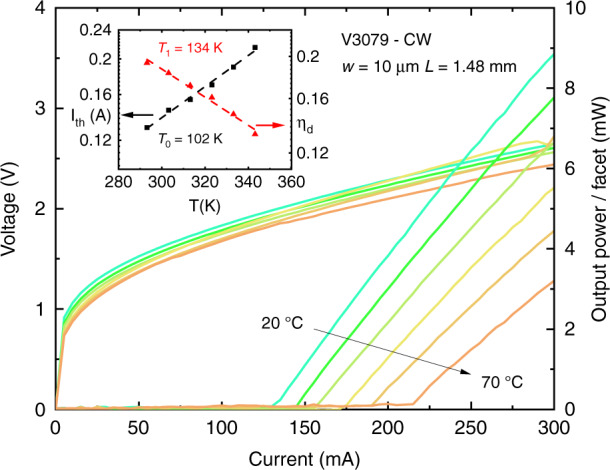
*L*–*I*–*V* curves in CW at various temperatures for a GaSb DL grown on a MOVPE GaAs-on-Si template. The inset shows the evolution of the current threshold and the external quantum efficiency as a function of the temperature. Adapted with permission from ref. ^107^. © Optical Society of America

As mentioned above (cf. section “Epitaxial growth of antimonide heterostructures on (001)Si substrates”), an interesting strategy in view of future large-scale production is to grow devices on III–V-on-Si templates prepared in separate growth runs. Growing GaSb DLs on MOVPE GaAs-on-Si templates is an attractive option since (i) these templates have been well developed for the growth of InAs/GaAs QDLs on Si^21,22,24,25^ and (ii) high-performance GaSb DLs grown on GaAs have been demonstrated^105,106^. We thus grew by MBE GaSb DLs on MOVPE GaAs-on-Si templates, similar to those used for InAs/GaAs QDLs, provided by Prof. Kei May Lau’s group at Hong Kong University of Science and Technology^83^. The residual substrate miscut was below 1° and the total thickness of the templates, including two sets of InGaAs/GaAs dislocation filtering layers, was ∼3 µm. Threshold current densities measured in pulsed conditions on broad-area DLs were around 400–450 A cm^−2^, whereas 8 × 1000 µm^2^ ridge devices exhibited CW threshold currents around 100 mA and 5–10 mW output power/facet at room temperature^107^. These DLs operated in CW up to 70 °C, limited by the experimental set-up, with a *T*_0_ around 100 K (Fig. 8). The performances of these DLs were comparable to those of similar GaSb DLs fully grown by MBE on Si substrates with a thinner buffer layer^37^.

**Fig. 9:**
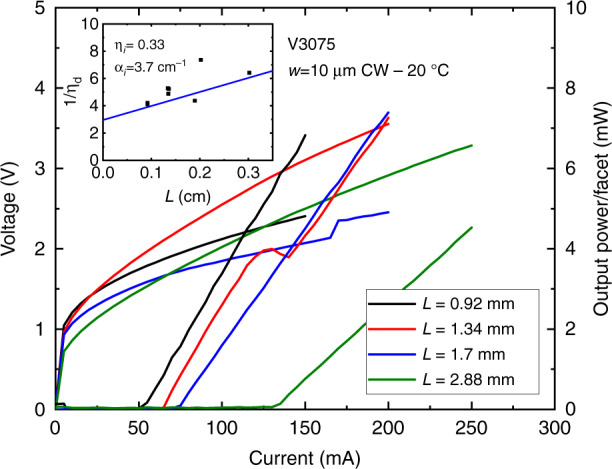
*L*–*I*–*V* curves in CW at RT for various GaSb DLs grown on a MOVPE GaSb-on-GaAs-on Si template. The inset shows the evolution of the inverse of the external quantum efficiency as function of the cavity length. Adapted with permission from ref. ^107^. © Optical Society of America

Another template included a ∼1 µm GaSb buffer layer with InAs/GaSb dislocation filtering layers grown on the preceding GaAs-on-Si template by MOVPE at Hong Kong University of Science and Technology^108^. The GaSb DLs grown by MBE on top of this composite template exhibited threshold current densities as low as 200–300 A cm^−2^ and CW threshold currents around 50–75 mA for 8 × 1000 µm^2^ ridge lasers (Fig. 9)^107^. These values are the best reported thus far for GaSb DLs grown on Si, at the price however of a very thick (5 µm) buffer layer. This resulted in occasional cracks after device processing, and sets a limit of around 10 µm to avoid crack formation with GaSb-based materials.

**Fig. 10:**
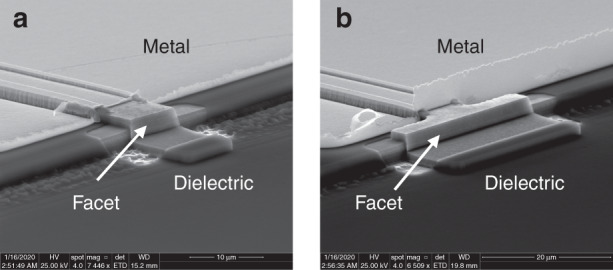
Scanning electron microscopy image. **a** Rectangular or **b** T-shape etched diode-laser facets. The different facet geometries were used to evaluate the impact of scattering at the facet edges. Adapted with permission from ref. ^109^. ^©^Optical Society of America

Finally, a set of DLs was grown on MOVPE GaSb-on-Si templates provided by Dr. T. Baron’s group at CNRS Grenoble, France^96^. These 500-nm-thick templates were grown on 300-mm Si wafers with a residual miscut around 0.11° and subsequently diced into 50-mm wafers for MBE growth of the DLs. Notably, etched-facet DLs of various geometries were fabricated (Fig. 10) and their performances were similar to those of cleaved-cavity DLs from the same epitaxial wafer, which is an important step toward PIC fabrication. Nevertheless, threading dislocation densities in the high 10^8^ cm^−2^ range resulted in high threshold current densities, in the 700–1000 A cm^−2^ measured with broad area DLs, whereas the CW threshold currents of 10 × 1000 µm^2^ devices were around 200 mA^109^.

### Summary

The results reported in sections “All-MBE grown GaSb-on-Si laser diodes” and “MBE-grown GaSb laser diodes on MOVPE-on-Si templates” above demonstrate that high-performance GaSb DLs can be obtained either by direct MBE of the whole structure on Si substrates or by MBE growth of the DL structure on GaAs-on-Si or GaSb-on-GaAs-on-Si templates obtained by MOVPE. This shows that large wafer-size MOVPE reactors and Si-microelectronics tools allow growing III–V buffer layers adapted to the subsequent epitaxy of GaSb DLs.

**Fig. 11:**
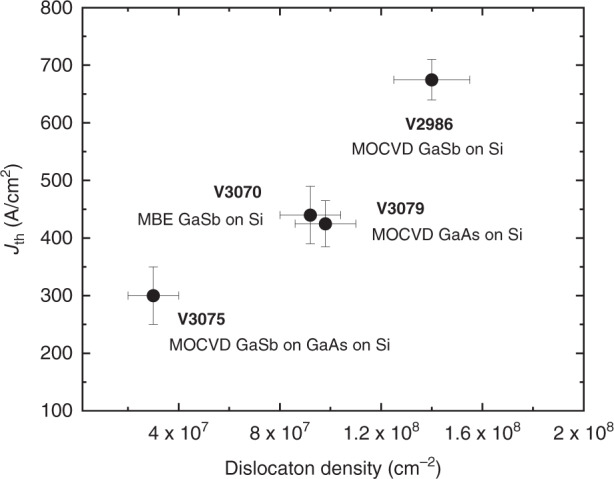
Evolution of the threshold current density measured at room temperature for GaSb DLs grown on various III–V-on-Si templates, as a function of the threading dislocation density estimated by atomic force microscopy. The nature of the buffer layer is indicated next to the experimental data points

The threshold current density of the GaSb DLs grown on III–V-on-Si templates appears to be sensitive to the threading dislocation density, as shown in Fig. 11 where we have plotted the threshold current densities of the various DLs discussed in the previous sections^110^. This finding is typical for DLs, and has also been observed with InAs/GaAs QDLs grown on Si in spite of their lower sensitivity to crystal defects^51^. On the one hand, this makes DLs perfect test vehicles to assess the structural quality of III–V-on Si templates and to track their progress^51^. On the other hand, this shows that dedicated strategies should now be implemented to decrease the threading dislocation density in GaSb DLs on Si, and thus improve their properties, as previously done with InAs/GaAs QDLs grown on GaP-on-Si templates^60,62^. Indeed, even the best values measured with GaSb DLs grown on Si (∼250 A cm^−2^, section “MBE-grown GaSb laser diodes on MOVPE-on-Si templates”) remain significantly higher than those achieved with similar DLs grown on native GaSb substrates, that are typically in the 70–150 A cm^−2^ range^28,98,99^, which should impact the device reliability. Figure 11 shows that dislocation densities in the mid-to-low 10^6^ cm^−2^ should allow reaching similar thresholds.

## GaSb-based type-II interband cascade lasers grown on Si

### ICL heterostructure

**Fig. 12:**
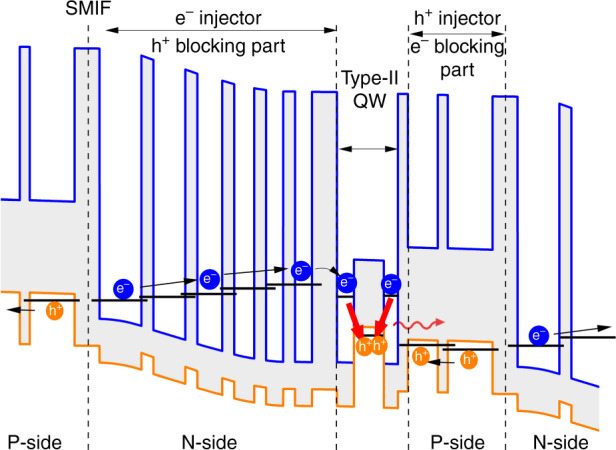
Band structure of a typical ICL. Adapted from ref. ^38^, used in accordance with the Creative Commons Attribution (CC BY) license

Besides the dislocation sensitivity mentioned above, GaSb DLs are limited to the 1.5–3.3 µm wavelength range, and their performance degrade seriously at a wavelength longer than 2.8 µm, due to Auger effects^111^. In this context, other band structures that rely on type-II radiative recombination have been proposed to extend the operation wavelength beyond 3 µm^29^. In the ICLs, the interband radiative transition occurs in a so-called “W” type-II QW made with a GaInSb hole-QW sandwiched between two InAs electron-QWs (Fig. 12)^112^. This “W” QW is surrounded by hole and electron injectors to feed the carriers into the QW. The injectors are composed of a series of alternating and graded thickness GaSb/AlSb and InAs/AlSb layers, respectively. The cascade scheme is achieved by stacking several periods of this layer sequence thanks to an InAs/GaSb semi-metallic interface (SMIF) that allows the electrons to tunnel into the conduction band of the next period (Fig. 12).

ICLs can be grown on InAs or GaSb substrates and they have progressively emerged as the most efficient MIR laser sources in the 3–5 µm wavelength range^30^.

### ICLs grown on Si

**Fig. 13:**
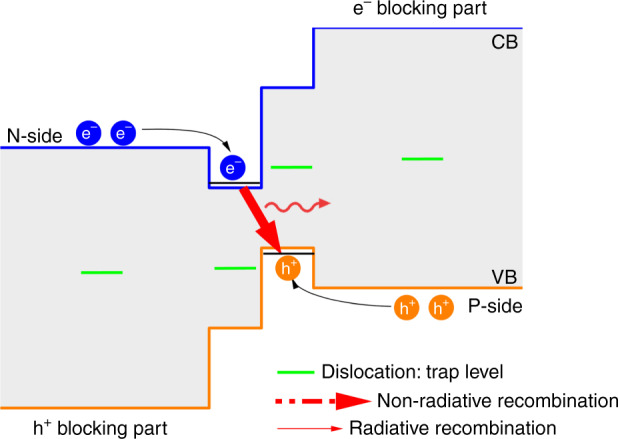
Representation of the recombination channels in an ICL. Adapted from ref. ^38^, used in accordance with the Creative Commons Attribution (CC BY) license

We have grown by MBE an ICL with a band-structure similar to that displayed in Fig. 12, scaled for emission around 3.5 µm on an on-axis GaSb-on-Si template grown by MBE^93,95^. The ICLs were processed with standard lithography and 8-µm wide ridge ICLs of different cavity lengths were tested in CW mode at various temperatures and compared to similar devices grown on the native GaSb substrate^38^. Strikingly, in spite of threading dislocation densities in the 10^8^ cm^−2^ range, the ICLs grown on Si exhibited performances very similar to their counterpart grown on GaSb, with threshold currents in the 30–70 mA for 1–3-mm-long cavities, and output powers around 20 mW/untreated facet. In addition, we performed a first device lifetime estimation with an ICL from this wafer. We have recorded >3800 h of operation in CW mode at 40 °C, which extrapolates to more than 30 years of device lifetime. Although this measurement has not been done under standard aging conditions, it demonstrates that ICLs are relatively immune to threading dislocations. We ascribe this finding to two peculiarities of the “W” type-II QW band structure. On the one hand, the defect levels are located outside of the radiative recombination path in the core of the active region (Fig. 13). On the other hand, the electron and hole injectors serve as barriers to the carriers, and prevent the electron and holes to escape in the *p*- and *n*-regions around the “W” QW, respectively (Fig. 13). As a consequence, non-radiative recombinations are also suppressed in the regions surrounding the QW. Altogether, this makes ICLs tolerant of defects.

After these promising results, more work is needed now to unravel the full potential of this integration technology, and to test other potential designs.

## InAs/AlSb quantum cascade lasers grown on Si

### QCL heterostructures

At still longer wavelengths than those covered by ICLs, i.e., at wavelengths longer than ∼5 µm, QCLs are the semiconductor lasers of choice^26^. Antimonides have a number of advantages for use in quantum-cascade lasers (QCLs) compared with other material systems. The conduction band offset between InAs and AlSb is much larger than in the GaAs and InP-based materials, so, high energies of intersubband (ISB) transitions can be obtained. For this reason, the InAs/AlSb system is well suited to short-wavelength QCLs and the shortest to-date QCL emission wavelength of 2.6 µm has been demonstrated with antimonide-based QCLs^113^. The small electron effective mass in InAs is another advantage of the InAs/AlSb system to obtain high ISB gain, which allowed achieving room temperature CW QCL operation at wavelengths up to 18 µm^114^. The first QCLs grown on silicon were demonstrated using antimonides^33^. These devices, emitting near 11 µm, exhibited performances comparable to those of the same lasers grown on native InAs substrates. However, the silicon substrate exhibited a 6°-miscut angle toward the [110] direction, not compatible with current industrial Si-photonics platforms.

The next attempt was made with InAs/AlSb QCLs emitting in the 7–8 µm region that is particularly interesting for sensing^1^. The active zone of these QCLs was based on vertical transitions in four coupled quantum wells and resonant phonon extraction. It contains 40 repetitions of the following layer sequence, in Å and starting from the injection barrier: **21**/69/**3**/56/**3.5**/54/**4.5**/51/**7**/48/**8**/46/**9**/46/**12**/42/**13**/39/**18**/37, where AlSb layers are in bold and the Si-doped layers (*n* = 6 × 10^16^ cm^−3^) are underlined. 2-μm-thick *n*-type InAs cladding layers formed the plasmon-enhanced dielectric waveguide, whereas undoped InAs layers inserted between the cladding layers and the active zone helped reduce the overlap of the guided mode with the doped material and thus reduced free-carrier absorption.

### InAs/AlSb QCLs grown on Si

As for the ICLs described in the previous section, an on-axis GaSb-on-Si template previously prepared by MBE^93,95^ was used as a virtual substrate for the MBE growth of the QCL (EQ609). For the sake of comparison, a similar QCL structure (EQ746) was grown under the same conditions in another growth run on a native InAs substrate. Atomic force microscopy (AFM) revealed the absence of an emerging antiphase domain even though the surface was rather rough (rms ∼9 nm). Preliminary transmission electron microscopy investigations revealed threading dislocations in the 10^8^ cm^−2^ range.

**Fig. 14:**
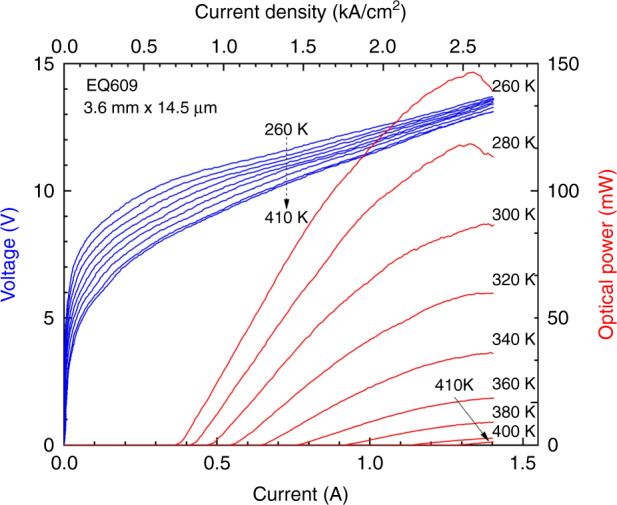
Voltage–current and light–current characteristics of a QCL grown on a Si substrate measured in pulsed mode at different temperatures. Reprinted from ref. ^34^, used in accordance with the Creative Commons Attribution (CC BY) license

**Fig. 15:**
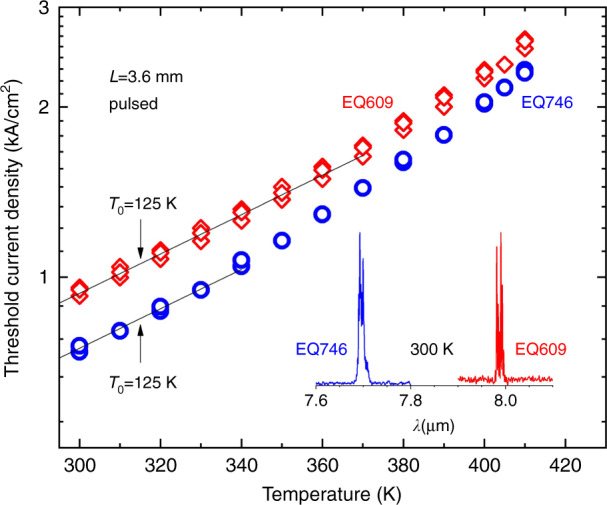
Threshold current density of QCLs grown on Si (red symbols, EQ609) and on InAs (blue symbols, EQ746) as a function of temperature and typical emission spectra. Reprinted from ref. ^34^, used in accordance with the Creative Commons Attribution (CC BY) license

Both sets of QCLs, grown either on Si or on InAs, operated in pulsed conditions up to a temperature of 410 K. Their threshold current density at RT was in the 920-950 A cm^−2^ range (Fig. 14) when grown on Si while it was around 750 A cm^−2^ for the reference devices grown on InAs (Fig. 15). The emission wavelengths were 7.7 and 8.0 µm at 300 K for the lasers grown on InAs and Si, respectively (inset in Fig. 15). The different emission wavelengths can be explained by the thermal strain in the structure, due to the difference in the thermal expansion coefficients of Si and the III–V materials, and by slight variations between different epitaxy runs.

**Fig. 16:**
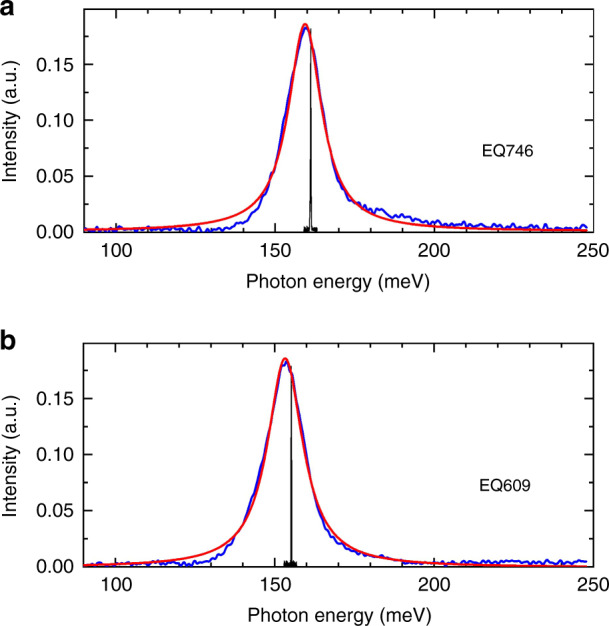
Emission spectra of the lasers measured at 300 K at a current density of 400 A cm^−2^ (∼0.5 *J*_th_) and above the threshold (black lines). **a** QCL grown on a native InAs substrate, **b** QCL grown on a Si substrate. Red curves show Lorentzian fits of the spectra. Reprinted from ref. ^34^, used in accordance with the Creative Commons Attribution (CC BY) license

Gain broadening or optical losses due to crystal defects can explain the slightly (∼25%) higher threshold current density for the QCLs grown on Si. To analyze this behavior, the emission spectra of the devices were recorded when driving them well below laser threshold (*J* = 400 A cm^−2^ ∼*J*_th_/2). The corresponding spontaneous emission spectra are displayed in Fig. 16 together with their respective laser spectra. Lorentzian fits of these data revealed full width at half maximum of 13.5 and 12.5 meV for the lasers grown on Si and on InAs, respectively. These values are very close, which indicates that the growth-related defects induced a negligible broadening of the gain curve. In fact, in InAs/AlSb QCLs, the impact on the radiative transition energy of symmetric thickness fluctuations of the AlSb and InAs layers is weak, and thus the gain curve is marginally affected^33^. Measurements on chips without cavities would however be necessary to definitely rule out any spectral narrowing induced by the resonator. Higher optical losses due to additional absorption on crystal defects are thus most likely to induce the increase in *J*_th_ of the QCLs grown on Si, as it is the case with DLs. The weaker degradation of short cavity length QCLs grown on Si fully supports this explanation^33^.

Still, these investigations reveal that InAs/AlSb QCLs grown on Si exhibit performances that are similar to those of comparable devices grown on their native, lattice-matched, InAs substrates, in spite of high threading dislocation densities^33,34^. The unipolar character of QCLs as well as their short radiative lifetimes are likely to explain much of this behavior. The poor performances of InP-based QCLs grown on Si could apparently contradict this conclusion^31^. However, the use of only binary compounds in InAs/AlSb QCLs makes their growth on Si less sensitive to defects than InP-based alloys^32^ and allows to fully benefit from QCL assets.

## Conclusion and perspectives

Following new achievements in the heteroepitaxy of III–V semiconductors on Si substrates, much progress has been made in the last decade on the epitaxial integration of semiconductor lasers, particularly in the near-IR wavelength range with InAs/GaAs quantum-dot lasers for data and telecom applications. There is, however, still a need for integrated lasers emitting at longer wavelengths, typically in the MIR range, for low-cost, low-size, portable sensing systems. Table 1 summarizes the relevant information for MIR semiconductor lasers integrated on Si through different techniques and based on different semiconductor technologies. Together with the details reported in this review article, this table shows that the integration of antimonide lasers emerges as the most promising route toward integrated MIR optoelectronics, even though it is still in its infancy. In addition, epitaxial lasers exhibit better performances than heterogeneously bonded devices (Table 1). GaSb diode lasers, the device of choice for an emission between 2 and 3 µm, exhibit threshold-current densities down to 250 A cm^−2^ and operate up to 80 °C without roll-over. Their performance, however, is sensitive to the residual dislocation density and remains lower than those of similar devices grown on native GaSb substrates. In contrast, at long wavelengths (>5 µm), InAs/AlSb quantum cascade lasers show similar performances to their counterpart grown on their native InAs substrates (threshold-current densities <1000 A cm^−2^ at room temperature, high *T*_0_). This probably arises from the unipolar character of QCLs and their short radiative lifetimes. At intermediate wavelengths (3–5 µm), the particular band structure of InAs/GaInSb interband cascade lasers appears to make them highly tolerant to threading dislocations. Device lifetimes over 30 years have even been extrapolated.

**Table 1:** Literature data for mid-IR lasers (*λ* ≥ 2 µm) integrated on silicon substrates, organized chronologically by laser technologies

Year	*λ* (µm)	Laser	Integration approach	Substrate	Threshold (A cm^−2^), operating mode, *T* (K)	*T*_max_ (°C)	Ref./section
2009	2.25	GaSb DL	MBE	4° offcut Si	5000, pulsed, RT	n.a.	^101^/“All-MBE grown GaSb-on-Si laser diodes”
2011	2	GaSb DL	MBE	7° offcut Si	900, CW, RT	35	^35^/“All-MBE grown GaSb-on-Si laser diodes”
2013	2	GaSb DL	BCB bonding	SOI	385, CW, RT	35	^12^/“Introduction”
2015	2	InP DL	Direct bonding	SOI	1500, CW, RT	35	^13^/“Introduction”
2016	2.3	InP DL	BCB bonding	SOI	2700, CW, 280	9	^14^/“Introduction”
2017	2.35	InP DL	BCB bonding	SOI	2900, CW, RT	20	^15^/“Introduction”
2020	2.3	GaSb DL	MBE	Si	400, CW, RT	>80	^37^/“All-MBE grown GaSb-on-Si laser diodes”
2021	2.3	GaSb DL	MBE DL on MOVPE buffer layer	Si	250, CW, RT	>80	^109^/“MBE-grown GaSb laser diodes on MOVPE-on-Si templates”
2018	3.6	GaSb ICL	Direct bonding	SOI	1100, pulsed, RT	50	^16^/“Introduction”
2021	3.5	GaSb ICL	MBE	Si	300, CW, RT	50	^38^/“ICLs grown on Si”
2016	4.8	InP QCL	Direct bonding	SONOI	1600, pulsed, RT	60	^17^/“Introduction”
2017	4.7	InP QCL	SU-8 bonding	SOS	5600, pulsed, RT	n.a.	^18^/“Introduction”
2018	4.4	InP QCL	MOVPE	6° offcut Si	1800, pulsed, 78 K	−30	^31^/“Introduction”
2018	11	InAs/AlSb QCL	MBE	6° offcut Si	1400, pulsed, RT	120	^33^/“QCL heterostructures”
2020	7.4	InP QCL	Direct bonding	SOI	2500, pulsed, RT	n.a.	^19^/“Introduction”
2020	8	InAs/AlSb QCL	MBE	Si	920, pulsed, RT	120	^34^/“InAs/AlSb QCLs grown on Si”

The last column indicates the original reference where the results have been published and the section where they have been mentioned and/or discussed in this review article

*DL* diode laser, *ICL* interband cascade laser, *QCL* quantum cascade laser, *SOI* silicon-on-insulator, *SONOI* silicon-on-nitride-on-insulator, *SOS* silicon-on-sapphire, *MBE* molecular beam epitaxy, *MOVPE* metal-organic vapor-phase epitaxy

These proofs of principle achieved, much work remains to be done in several directions before the emergence of fully integrated sensors.

First, we have seen above (cf. section “Summary”) that DLs are clearly sensitive to dislocations whereas ICLs (section “ICLs grown on Si”) and QCLs (section “InAs/AlSb QCLs grown on Si”) are much less sensitive. However, these devices do not cover the MIR range below 3 µm. The threading defect density thus needs to be mitigated through optimization of the buffer layer growth and structure, as demonstrated in the near-IR^63^, to improve the DL performance. Quantum dots however are not a straightforward option since the InSb/GaSb material system intrinsically gives rise to low-radiative efficiency QDs^115,116^. Laser designs based on the ICL concept could possibly be applied to other materials systems exhibiting comparable band-structure configurations, opening the way to shorter wavelength applications^38^. All these developments should be coupled to standard device lifetime evaluation and modeling to fully qualify these MIR integrated lasers.

Regarding laser performance, there are no universal specifications for sensing applications. Depending on the detection technique, the species to be detected, the target sensitivity, and the application field, MIR lasers for sensing can operate in pulsed—or in CW—mode close to room temperature. They must, however, be single mode and tunable over a few nanometers^117^, which requires regulating their temperature. Single-mode emission can be achieved by defining gratings either onto the III–V semiconductor^28^ or on the Si photonics platform^15^ to obtain distributed-feedback lasers. Similarly, there are various possibilities for the detection part of the sensor. Photodetectors can also be grown on Si^118,119^, or the same heterostructure can be used as a laser and detector on different parts of the wafer^120,121^.

Finally, the epitaxial integration of lasers—whether near-IR or MIR lasers—has so far been limited to the demonstration of discrete devices. The next step will be to integrate these devices with photonic functions, which requires coupling the light with passive waveguides. This is a challenge in the epitaxial approach since the thick buffer layers (>1 µm) and the high defect density near the III–V/Si interface make it difficult to use tapers or grating couplers to force the mode down into a waveguide. Other options such as butt coupling, evanescent coupling, or slotted ridges to incline the emission downward^22,122–124^ have been proposed. The light coupling strategy in the epitaxial integration scheme remains to be explored, whatever the wavelength range and target application.

Most of these research routes are being investigated in various laboratories around the world, and we are confident that mid-infrared integrated sensors will emerge in the not-so-distant future.
